# Supplementary material for: A UMLS-based spell checker for natural language processing in vaccine safety
Source: BMC Med Inform Decis Mak. 2007 Feb 12;7:3. doi: 10.1186/1472-6947-7-3 (PMC1805499; doi:10.1186/1472-6947-7-3)
Supplement: Additional file 13 — RAP application directory. Sets up directory for RAP (RDF application for PHP) source code [file 1472-6947-7-3-S13.gz › rap/doc/phpdoc/__filesource/fsource_dataset__datasetIteratorFindQuadsDb.php.html]

xml version="1.0" encoding="iso-8859-1"?


File Source for IteratorFindQuadsDb.php


# Source for file IteratorFindQuadsDb.php

Documentation is available at IteratorFindQuadsDb.php

```
1. <?php
2. /**
3. * ----------------------------------------------------------------------------------
4. * Class: IteratorFindQuadsDb
5. * ----------------------------------------------------------------------------------
6. *
7. * @package     dataset
8. */


11. /**
12. * Implementation of a quad iterator.
13. *
14. * This Iterator should be used like:
15. * for($iterator = $dataset->findInNamedGraphs(null,null,null,null); $iterator->valid(); $iterator->next())
16. * {
17. *    $currentQuad=$iterator->current();
18. * };
19. *
20. * <BR><BR>History:
21. * <LI>05-30-2005                : First version of this class.</LI>
22. *
23. * @version  V0.9.1
24. * @author Daniel Westphal (http://www.d-westphal.de)
25. *
26. *
27. * @package     dataset
28. * @access    public
29. ***/
30. class IteratorFindQuadsDb
31. {
32. /**
33. * Holds a reference to the associated DB resultSet
34. * @var        $dbResultSets ADODB result
35. * @access    private
36. */
37. var $dbResultSet;

39. /**
40. * Holds a reference to the associated datasetDb
41. * @var        $datasetDb datasetDb
42. * @access    private
43. */
44. var $datasetDb;

46. /**
47. * boolean value, if the results should be returned as triples
48. * @var        boolean
49. * @access    private
50. */
51. var $returnAsTriples;

53. /**
54. * Constructor.
55. *
56. *
57. * @param dataset
58. * @access    public
59. */
60. function IteratorFindQuadsDb(&$dbResultSet,&$datasetDb,$returnAsTriples=false)
61. {
62. $this->dbResultSet=& $dbResultSet;
63. $this->datasetDb=& $datasetDb;
64. $this->returnAsTriples=$returnAsTriples;
65. }

67. /**
68. * Resets iterator list to start
69. *
70. * @access public
71. */
72. function rewind()
73. {
74. //not supported
75. }

77. /**
78. * Says if there are additional items left in the list
79. *
80. * @return    boolean
81. * @access    public
82. */
83. function valid()
84. {
85. if (($this->dbResultSet ===false) OR ($this->dbResultSet->EOF) )
86. return false;

88. return true;
89. }

91. /**
92. * Moves Iterator to the next item in the list
93. *
94. * @access    public
95. */
96. function next()
97. {
98. if ($this->dbResultSet!==false)
99. $this->dbResultSet->moveNext();
100. }

102. /**
103. * Returns the current item
104. *
105. * @return    mixed
106. * @access    public
107. */
108. function &current()
109. {
110. if ($this->dbResultSet===false)
111. return null;
112. // subject
113. if ($this->dbResultSet->fields[5] == 'r')
114. $sub = new Resource($this->dbResultSet->fields[0]);
115. else
116. $sub = new BlankNode($this->dbResultSet->fields[0]);

118. // predicate
119. $pred = new Resource($this->dbResultSet->fields[1]);

121. // object
122. if ($this->dbResultSet->fields[6] == 'r')
123. $obj = new Resource($this->dbResultSet->fields[2]);
124. elseif ($this->dbResultSet->fields[6] == 'b')
125. $obj = new BlankNode($this->dbResultSet->fields[2]);
126. else {
127. $obj = new Literal($this->dbResultSet->fields[2], $this->dbResultSet->fields[3]);
128. if ($this->dbResultSet->fields[4])
129. $obj->setDatatype($this->dbResultSet->fields[4]);
130. }

132. if($this->returnAsTriples)
133. return (new Statement($sub, $pred, $obj));

135. return (new Quad(new Resource($this->dbResultSet->fields[7]),$sub,$pred,$obj));
136. }

138. /**
139. * Returns the key of the current item
140. *
141. * @return    integer
142. * @access    public
143. */
144. function key()
145. {
146. return $this->dbResultSet->_currentRow;
147. }
148. }
149. ?>
```

Documentation generated on Thu, 7 Jul 2005 13:41:47 +0200 by phpDocumentor 1.3.0RC3
